# Rules of collective migration: from the wildebeest to the neural crest

**DOI:** 10.1098/rstb.2019.0387

**Published:** 2020-07-27

**Authors:** Adam Shellard, Roberto Mayor

**Affiliations:** Department of Cell and Developmental Biology, University College London, Gower Street, London WC1E 6BT, UK

**Keywords:** neural crest, collective migration, contact inhibition of locomotion, supracellular, co-attraction, alignment

## Abstract

Collective migration, the movement of groups in which individuals affect the behaviour of one another, occurs at practically every scale, from bacteria up to whole species' populations. Universal principles of collective movement can be applied at all levels. In this review, we will describe the rules governing collective motility, with a specific focus on the neural crest, an embryonic stem cell population that undergoes extensive collective migration during development. We will discuss how the underlying principles of individual cell behaviour, and those that emerge from a supracellular scale, can explain collective migration.

This article is part of the theme issue ‘Multi-scale analysis and modelling of collective migration in biological systems’.

## 1. Collective migration

The annual migration of the great wildebeest in the Serengeti, a group of malignant cancer cells escaping into a blood vessel, bacteria swarming over solid surfaces to produce a biofilm: all of these are examples of collective behaviour, which refers to the phenomenon that an individual unit's actions are dominated by the influence of others. More specifically, these are all examples of collective migration, which is defined by the movement of groups whereby individuals both move in concert with one another and affect each other's behaviour. This differs from individual migration, whereby movement is undertaken solitarily, and individuals do not influence each other. The interactions between individuals migrating collectively leads to emergent behaviour. For instance, birds show patterns of movement that are only achieved when they are part of a flock [1], and swarming bacteria perform large-scale swirling and streaming motions which are not seen during individual bacterial migration (which instead, move by swimming) [2,3].

**Figure 1.**
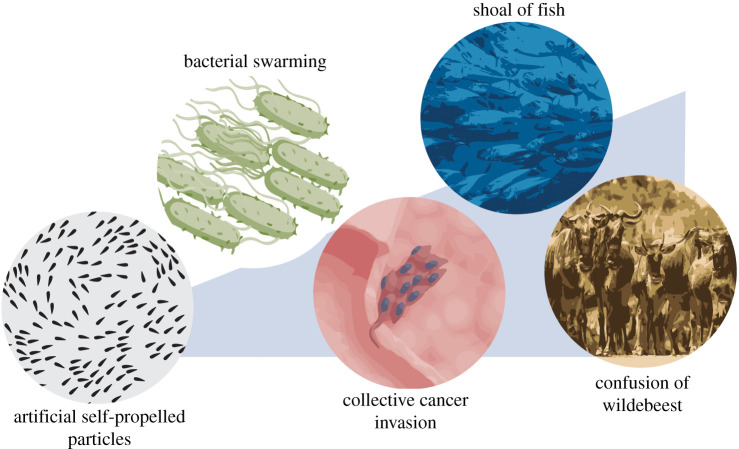
Collective migration at all scales. Collective migration is found at practically all levels, from self-propelled particles to bacteria, cancer and animals.

Collective migration can offer distinct advantages over solitary migration. The swarming behaviour of bacteria, whereby movement of multicellular bacterial aggregates is powered by rotating flagella, optimizes the search for nutrients and other necessary resources [4]. Most invasive cancers infiltrate collectively [5], which defines malignant function, and cancer aggression is likened more to collective cell migration than to single-cell migration [6]. At the other end of the spectrum, animals like the great wildebeest must relocate to new sources of food; their enormous herd size providing protection and a greater chance of survival from predators during their migration. Collective motion also appears as an emergent trait in artificial self-propelled particles [7] and other non-living systems. Thus, collective migration is evident at practically all scales (figure 1), in both natural and artificial systems.

The universality of this phenomenon [8] has led to intense investigation; collective migration has been studied substantially in animals [1,9], but only comparatively recently in cells. The fact that collective motion is conserved at different scales suggests that there may be common, underlying principles that govern the movement of groups. It has long been noted in animals that there is a remarkable degree of long-range organization that cannot be apparent to each individual, meaning there must be some degree of leadership or communication [10]. Likewise, embryonic morphogenesis involves large-scale tissue movements that presumably require cells to direct one another because communication and influence are necessary to organize large groups.

## 2. Rules for collective migration

Altogether, the combined empirical evidence and computational simulations of collective motion of different entities suggests that three ‘rules’ are sufficient to describe and explain collective movement: repulsion, attraction and alignment (box 1).

Box 1. **Definitions of the three rules of collective migration.**

*Attraction*: a behaviour that causes individuals to steer towards the centre of mass, which is the average position of individuals within a certain radius.

*Repulsion*: the behaviour that causes individuals to steer away from all its neighbours.

*Alignment*: a behaviour that causes an individual to line up with individuals close by, such that it moves with the averaged heading of the nearby individuals.

Firstly, repulsion, whereby individuals separate to avoiding crossing neighbours, is exhibited between all individuals of the group. Individuals cannot exist in the same space as one another; rather, they are loosely connected. Secondly, the group's cohesion is driven by individuals being attracted to one another, which means that individuals steer towards the average position of neighbours, which ensures that the group migrates collectively rather than individually. Repulsion is finely balanced with attraction to maintain the integrity of the group as a loose collective [11]. Thirdly, the movement of neighbouring individuals is aligned, meaning they coordinate motion and move in the same direction (the average heading of neighbours). For example, starlings decide their orientation from only the six or seven closest birds [12]. When neighbour velocities are not aligned, the group becomes disorganized and collective motility is impaired. The ubiquity of these ‘rules’ has led to intense investigation. For example, these principles form the basis of a multiplicity of mathematical models that are used to study collective motion. One of the first simulations of collective motion was an artificial program called Boids, which simulates the flocking behaviour of birds [13]. Since then, the principles of swarm intelligence, the collective behaviour of self-organized systems, has even been employed in artificial intelligence, such as robotics [14]. In this review, we will focus on how these rules work during collective cell migration, and how they fit with phenomena that emerge at a higher level: that of the whole migratory group.

## 3. Collective cell migration: epithelial versus mesenchymal cells

**Figure 2.**
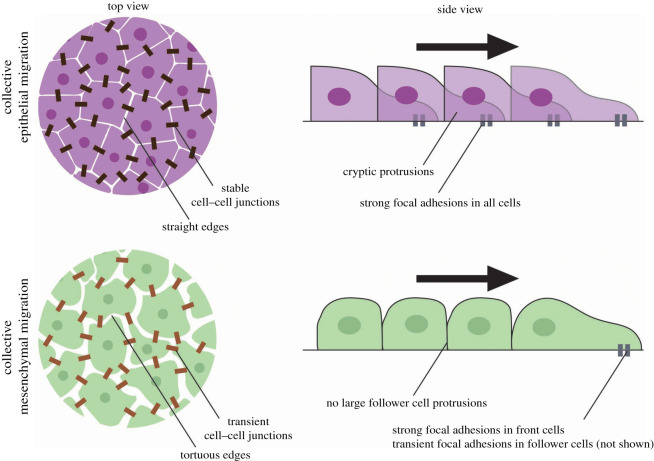
Collective migration of epithelial cells and mesenchymal cells. Epithelial migration can arise from an unjamming transition of quiescent epithelial sheets. Such unjammed, motile epithelia display packs of collectively migrating cells (purple cells), while maintaining strong intercellular junctions (dark brown rectangles) and epithelial markers, such as E-cadherin. Epithelial migration is also evident in wound healing. Leader cells tend to form large forward-facing protrusions, with follower cells also contributing significant traction forces to move the sheet forward (teal rectangles). Mesenchymal migration can arise from an epithelial-to-mesenchymal transition, in which cells (green cells) lose apicobasal polarity in favour of front-rear polarity, and intercellular adhesions become weaker and more transient (light brown rectangles), which is associated with a change in gene expression, such as E-cadherin being replaced by N-cadherin. Whereas leader cells form strong focal adhesions, follower cells do not. The black arrow indicates the direction of migration.

Collective cell movements underly many developmental and pathological processes including embryonic morphogenesis, wound healing and diseases like cancer invasion [15,16]. During collective cell migration, many cells move together, cooperatively and coordinately, in a manner similar to that of animals. To a large degree, the behaviours exhibited depend on the type of cells that are moving: collective migration events can involve epithelial sheets with cells retaining apicobasal polarity markers, including strong, stable intercellular junctions; alternatively, they can involve the cooperative interaction between looser mesenchymal cohorts mediated by transient adherens junctions (figure 2) [16].

Epithelia are normally quiescent, with cells ‘jammed’ in their respective positions, lacking the energy to overcome high junctional tension. Fluidization of the tissue via an unjamming transition in which tension is reduced [17] permits collective motility of large epithelial groups, such as those cells involved in wound healing [18] or cells from asthmatic airway epithelium [19]. During such large-scale movements, cell adhesion is high, and the group remain tightly bound, and all cells contribute equally to the group's movement [20–24]. For example, leader and follower cells produce protrusions oriented in the direction of migration, and both produce traction forces that pull on the substrate [23,25–27]. The arrest of cellular monolayers can also be driven by strong cell–cell binding [28].

By contrast, mesenchymal cells, such as the mesendoderm (also called the prechordal plate) or neural crest, display front-rear polarity with weaker, more transient cell contacts, which may redirect protrusion formation contributing to the overall directionality [16]. This allows them to migrate as a loosely connected pack [29]. Mesenchymal migration is, therefore, far more akin to the type of collective motion observed in animals, bacteria and synthesized self-propelled particles than epithelial migration is. Consequently, the three conserved ‘rules’ of collection motion are likely to be more applicable to mesenchymal migration than to epithelial migration.

Surprisingly, the concepts that emerge from the study of cells reveal that the types of interactions, behaviours and movements are like those of collectively moving animals. Repulsion between cells arises as a consequence of the formation of cell–cell contacts between individuals, and it has been observed in the collective migration of mesenchymal cell populations like the neural crest [30–32], the mesendoderm [33,34] and cancer cells [35–37]. By contrast, in epithelia, repulsion does not occur as cells are tightly held together by strong intercellular junctions.

Group cohesion arises when cells express both a ligand and its receptor. This leads to cells being mutually attracted to each other [38,39]. For example, *Dictyostelium* amoebae secrete the chemoattractant cyclic AMP (cAMP), which encourages aggregation [40], whereas mutual cell attraction is not required in epithelial cells because they maintain strong intercellular adhesions which keep them together.

Finally, in all the cases, cell motion is aligned [41–43]. For instance, *Xenopus* axial mesoderm cells move in the same direction as one another [43]. Surprisingly little is known about the mechanisms governing cell alignment, despite it being essential for collective motion. In some cases, such as the collective migration of keratinocytes *in vitro*, alignment is partially a consequence of each individual cell responding to growth factor signals, although this also does not totally account for their coordinated alignment [44]. Likewise, the orientation of daughter cells following mitosis can form locally ordered regions [45], but this does not explain the aligned motion of non-proliferative cells. Instead, coordination of motion requires direct or indirect communication between cells of the migrating group.

Various models of self-propelled particles and inelastic collisions between particles have demonstrated that collision properties affect alignment [46–49]. The collision properties of certain malignant cancer cells also mediate the alignment of cell motion. Cells at the edge of the group have more propulsion than those in the centre because they experience less contact inhibition of locomotion—a mechanism of cell repulsion that repels cells away from cell–cell contacts—which leads to edge cells having stronger alignment interactions compared with those at the centre of the cluster [50].

In fibroblasts, cell collision guidance, which refers to the reorientation of cells to one another after collision, is vital for generating long-range alignment patterns in a mechanism that involves suppression of the actomyosin machinery at the cell contact [51]. A consequence of this behaviour is the generation of an anisotropic extracellular matrix (ECM) [51], which then feeds back to cell alignment. For instance, contact guidance, which refers to the response of cells to the underlying substrate or hard boundaries, can mediate the orientation of cells [52,53]. Thus, mutual alignment between fibroblasts and underlying collagen fibres produces a long-range ordered cell pattern [51,54].

The surge of research into collective migration over the past decade has led to various simulations being developed. Computational models that simulate collective cell migration in a variety of morphogenetic processes have also shown that, like for animal migration, repulsion is needed [55,56]. Likewise, spontaneous emergent coordinated patterns of movement, such as streaming and swirling, arise in models as a consequence of the inherent cellular behaviours involved in contact-dependent repulsion, such as cell repolarization [57–62].

Although collective migration can be simulated in various ways, the enormous availability of quantitative biological data on cells means quantitative mathematical models which incorporate experimental values can be of great benefit to understanding the relationship and relative importance of various parameters, such as attraction, repulsion and alignment. The quantitative information from cell dimensions, cell trajectories (such as velocity and persistence) and gene expression are all parameters that can be incorporated into models to test their relative contribution to collective behaviour. For example, quantitative modelling was important in concluding that although aligned fibroblasts are more persistent in their movement, this was insufficient to drive alignment and lead to anisotropic ECM [51].

Interestingly, many different experimental and theoretical studies have described that directional movement by chemotaxis is enhanced when cells cooperate as a group compared to if they responded as individuals, including neural crest cells, lymphocytes and breast organoids [63–66]. Thus, theory and empirical observations have shown that these simple interaction rules are sufficient to generate the mechanisms responsible for emergent group migratory properties and highly efficient collective behaviour [1,67].

## 4. Collective migration of neural crest cells

**Figure 3.**
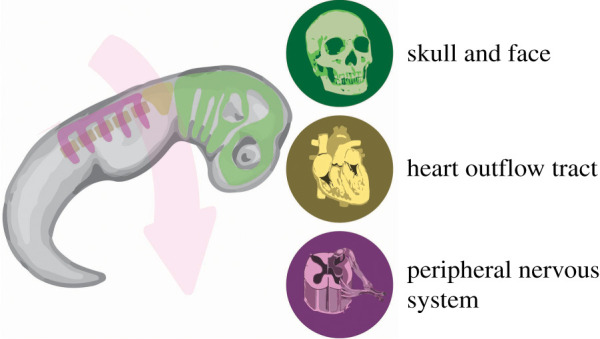
Neural crest migration. The neural crest form at the border of the neural plate (top of the embryo), and then collectively migrate (pink arrow) toward the pharyngeal arches (bottom of the embryo). The green areas correspond to cranial neural crest migration, the yellow to cardiac neural crest migration, and the purple to trunk neural crest migration, which must move over the somites (brown ovals). When they reach the pharyngeal arches, they differentiate into a variety of cell types and contribute to many tissues and organs, including the craniofacial structures, the outflow tract, dorsal root ganglia and the enteric nervous system.

Many of the cellular mechanisms responsible for controlling these ‘rules’ have been discovered in a model system for collective mesenchymal migration: the neural crest [68]. The neural crest is a multipotent stem cell population of vertebrate embryos. It is initially formed from the ectoderm, at the border of the neural plate. It then delaminates and undergoes extensive migration to colonize the pharyngeal arches on the other side of the embryo, where they contribute to many different tissues (figure 3) [69]. Different subpopulations of neural crest arise from and inhabit different regions along the embryo. For example, the cranial neural crest contributes most of the tissues that make up the craniofacial structures, including bones of the skull, cartilage and connective tissue of the nose and the underlying nerves.

Most neural crest migrate collectively as chains, in streams or as sheets [68,70], in a manner that has been likened to invasive cancers [71,72]. For example, cephalic neural crest cells maintain short and long-range cell–cell interactions [73]. Like other cell types that have the capacity to migrate both collectively and individually, overall movement is faster and more directional when the cells migrate as a group [64,65], which indicates that interactions between individuals of the group promote directionality [73]. Interestingly, the emergence of improved efficiency of motion in collectives, compared to individuals, is also evident at other scales; collective bacterial swarming is more efficient than solitary swimming [4,74]. Similarly, chicks of king penguins, larval damselfish and pigeons all move straighter, faster and via more efficient routes when moving as a group rather than alone [1,75–78].

**Figure 4.**
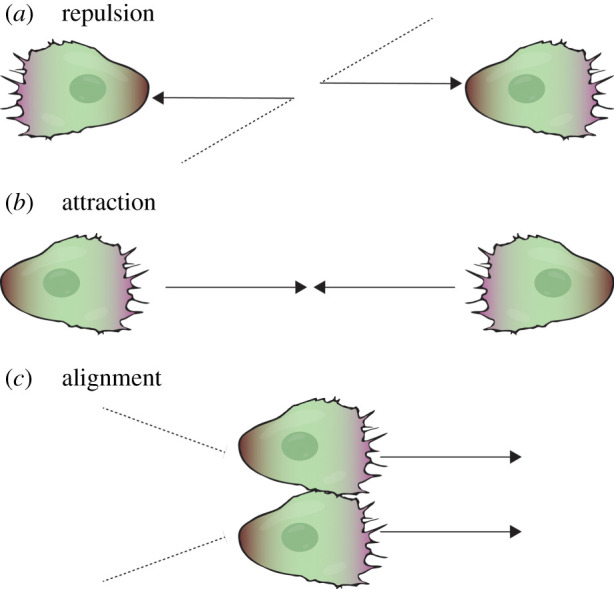
Cellular mechanisms of the three ‘rules’ of collective migration. (*a*) Two colliding cells (black dotted lines) repolarize and move away from each other (black arrows) by contact inhibition of locomotion. Purple regions represent the local activity of Rac1 at the leading edge and brown regions represent the local activity of RhoA at the cell rear. (*b*) Two cells are mutually attracted by C3a-dependent chemotaxis. (*c*) Two colliding cells move together for a short period of time after colliding, and before repulsion. This is one mechanism that drives the alignment of cell motion.

Repulsion between neural crest cells is mediated through contact inhibition of locomotion (CIL; figure 4*a*), which refers to the phenomenon by which colliding cells repolarize and move away from each other [79,80]. CIL is the driving force for the collective cell migration of many different cell types [30,80–82]. The mechanism by which CIL occurs depends on the type of migratory cohort, but in the case of the neural crest it involves N-cadherin, ephrins and planar cell polarity signalling from sites of cell–cell contact, which leads to high levels of RhoA at the contact and high levels of Rac1 away from the contact [30,31,83]. These small GTPases regulate adhesive forces such that cells pull away from each other [84].

The attraction between neural crest cells is mediated by short-range chemotaxis (also called co-attraction; figure 4*b*). Each cell within the group expresses both chemoattractant, C3a, and its cognate receptor, C3aR, meaning cells are attracted to each other [38]. A balance between the repulsive and attractive mechanisms is essential for collective migration, ensuring the neural crest is maintained as a loosely connected migratory group, which behaves in a fluid-like manner [85]. When the attraction is too strong, the group fails to move; when repulsion is too strong, the cells disperse away from each other, and migration becomes individualistic rather than collective [11,38,86].

Finally, cell tracking and particle image velocimetry has revealed that there is alignment of motion during collective neural crest migration, with neighbouring cells tending to move in the same direction at a similar speed (figure 4*c*) [41,42]. The mechanisms governing aligned cell movement are unclear, but likely involve space limitations and the fact that cells are adhered to one another. Also, N-cadherin-dependent adhesion junctions form between colliding cells, meaning that through physical attachment, cell movement is aligned until the repulsion stage is reached [79]. This is further supported by theoretical and experimental evidence, which shows that the emergence of persistently polarized collective cell movements when they are under confinement can arise from CIL between colliding cells [59]. *Dictyostelium* cell alignment in collective migration is, in part, directed by the induction of a new leading edge from cell–cell contact and the accompanying forward protrusion, which has been called contact activation of locomotion (or contact following of locomotion) [39,87], indicating that the effects of cell collision may be more multifaceted than initially thought.

## 5. Directional collective migration requires external cues

Unlike the motion of some animal groups, like many shoals of fish, or synthetic self-propelled particles, collective cell migration normally displays persistent long-range directionality. However, whereas collective motion emerges from the ‘rules’ outlined above, when cells are confined, overall net displacement does not, because these ‘rules’ alone do not confer front-rear polarity on the group, nor necessarily a mechanism by which to move in a persistently directed manner. This implies that there are mechanisms of guidance. For the neural crest, it is the cell's interaction with the surrounding environment that directs their persistent directional movement. The directionality of neural crest migration is dependent on collective chemotaxis [64], which refers to the movement of a cell group along a gradient of soluble chemical cues. Various chemokines and growth factors have been identified for neural crest migration in different species and subpopulations [88]. In *Xenopus* and zebrafish, placodal cells, which give rise to structures of the sensory nervous system, secrete the chemokine SDF1 (also called CXCL12), which attracts cranial neural crest cells [89]. The neural crest expresses its cognate receptor, CXCR4, causing it to ‘chase’ after the placodes by collective chemotaxis. CIL mediates repulsion between the cell populations, meaning the placodal cells ‘run’ away [89]. This ‘chase and run’ mechanism results in the directional movement of both populations. Computational and experimental evidence supports the idea that collective migratory streaming exhibited by the neural crest is an emergent property based on the combined interactions of neural crest cells with each other and with the placodes [90]. Chemotaxis similarly mediates the directional migration of most of the other collectively migrating cell populations, including border cells [91], the posterior lateral line primordium [92], tracheal cells during branching morphogenesis [93,94] and endothelial cells during angiogenesis [95]. Non-artificial systems of collective migration also rely on external signals to direct motion; bacterial swarming is a chemotactic response to nutrient gradients, and many animal groups move along food gradients [96,97].

A variety of other mechanisms also play a role during neural crest migration [98]. Repulsive signals, including ephrins, semaphorins, extracellular matrix molecules like versican and the BMP antagonist, DAN, exist between the neural crest to confine them into streams, promoting directional migration and preventing ectopic neural crest invasion [86,98–101]. Mechanical signals of mesoderm stiffness are sensed by the overlying neural crest to controls its migration; it can only migrate when the mesoderm is rigid and not when it is soft [102], meaning both chemical and mechanical signals control collective neural crest migration. Confinement of neural crest cells also promotes directional persistence by optimizing density based on the parameters of CIL and co-attraction [86]. These mechanisms all work together to regulate neural crest cell migration [103], but they are not exclusive to the collective migration of neural crest: sensing of mechanical signals, chemorepellents and confinement also control collective cell migration in other systems [104–107], and external attractive and repulsive cues are easily analogous with collective movement at other scales.

## 6. Supracellular mechanism of collective chemotaxis

At all scales, although the three rules of attraction, repulsion and alignment can generate collective motility, they are insufficient to explain persistent directional collective migration; instead, external signals are required to direct movement. For the neural crest, collective chemotaxis to placodal cells secreting SDF1 is essential in determining directionality [64,89,90]. The mechanism by which the cranial neural crest of *Xenopus* and zebrafish undergo collective chemotaxis has been recently elucidated; this shows that the neural crest move by supracellular migration, which is a type of collective migration whereby movement can be better described by the behaviour and activity of the group as a whole rather than by the individuals of which it is comprised [108].

**Figure 5.**
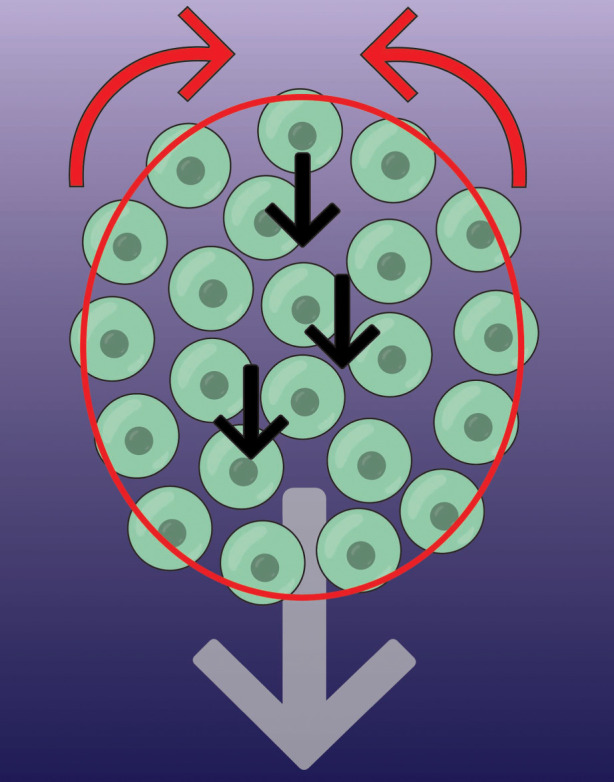
Supracellular migration of the neural crest. A group of neural crest cells (green circles) migrate forward by chemotaxis (grey arrow; chemotactic gradient is purple background). The mechanism for this directed movement relies on a supracellular contraction force at the rear (red arrows), driven by a pluricellular actomyosin cable (red). Contraction is inhibited by chemoattractant at the front. This rear force causes cells to intercalate forward (black arrows), moving to the front before becoming mechanically connected at the edge.

Neural crest cells at the edge of the group are mechanically connected by a multicellular actin cable, seemingly via N-cadherin adherens junctions across cell contacts [42]. The actin cable is associated with the motor protein, non-muscle myosin II, which exerts contractile force synchronously between adjacent cells at the rear of neural crest groups. At the front, contraction is inhibited by neural crest chemoattractants such as SDF1 or PDGF [42]. This contraction forces cells to intercalate forwards from the rear, with a knock-on effect causing those cells in front of them to move forward (figure 5). Overall, this causes cells in the group to flow forward, and they do so with liquid-like behaviour thanks to loose N-cadherin-dependent cell adhesions that are rapidly turned over [85]. Thus, a physical supracellular mechanism coordinates long-range collective chemotaxis of neural crest cells.

Alongside contractile forces traction forces also contribute to the directed migration of collective chemotaxis. Cells at the free edge have larger protrusions than those in the centre [83], and SDF1 enhances Rac1 activity in leading cells [64], meaning traction forces are higher at the front than anywhere else. High traction at the front and high contraction at the rear likely work together to move the group forward, in a similar way to how single cells move [109], therefore, making the cluster act like a ‘supracell’ [108].

## 7. Integrating individual rules with supracellular behaviour

**Figure 6.**
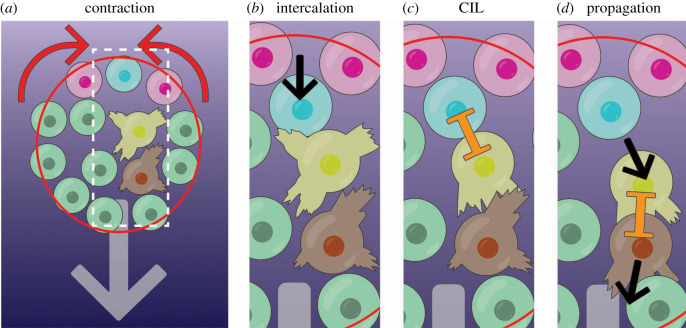
Stages of supracellular neural crest cell migration. (*a*) Cells at the edge of the cluster are linked by an actomyosin cable (red). The cable contracts at the rear (red cable, red arrows indicate contraction) but not at the front. (*b*) All cells at the rear are brought closer together, causing cells to intercalate forwards (blue cells intercalation as pink cells move closer together; movement is black arrow). (*c*) The intercalating cell (blue) makes contact with the unpolarized cell in front (yellow), causing it to polarize, producing protrusions forward. This occurs by CIL (orange inhibition symbol). (*d*) This cell (yellow) then moves forward, propagating the signal to the cells in front (brown cell), and so forth. Thus, an anterograde wave of aligned forward cell flow emanates from the rear of the cell cluster.

How do the three rules that govern collective cell migration (i.e. attraction, repulsion and alignment) espouse those at the supracellular level? Supracellular contractile forces make neighbouring neural crest cells come closer together (figure 6*a*) [42]. These forces cause cells to intercalate forward from the rear (figure 6*b*, blue cell). This intercalation at the rear of the cluster moves this rear cell forward, increasing the contact with the cell immediately in front of it which was unpolarized (figure 6*b*, yellow cell). This increased contact leads to a CIL response in the front cell favouring its forward polarity (figure 6*c*, yellow cell). This polarized cell will move forward, contacting other cells and inducing the same cycle of polarization by CIL leading to the propagation of a forward wave of movement [42] (figure 6*d*). Interestingly, the movement of edge cells into the middle was also observed and modelled in collective malignant cancer cell chemotaxis [50].

The mechanism of supracellular contractility, therefore, complements the rules of collective motion. In the absence of an actomyosin cable, the neural crest are motile but do not display chemotaxis, which indicates it is required for directed movement but not cluster motility [42]. Supracellular contractility supports co-attraction, by forcing cells to move together. In doing so, colliding cells form cell adherens junctions and often move together before the repulsion phase of CIL. Thus, contractility promotes the alignment of neighbouring cell motion by promoting CIL to generate directed flow. This enhancement of CIL may explain why clusters of neural crest cells are more persistent and directional than cells that move alone [64]. Cell flow is also dependent on the group's low intercellular adhesion strength [83,85]. In support of this idea, mathematical modelling has shown that collective movement can be heavily modulated by changing CIL parameters. Finally, the supracellular polarity of traction forces likely also contributes to aligned cell movement in the group [83,84].

The role for supracellular behaviour may be different in other systems. However, similar to the neural crest, the actomyosin network is highly organized across epithelial sheets during wound healing, including a supracellular actomyosin cable at the wound edge that is required for coordination of cell movements and to prevent scarring [110,111].

## 8. Conclusion

There is substantive evidence that repulsion, attraction and alignment are sufficient for the collective movement of animals, and equivalent behaviours have been described for the collective migration of many cell types, especially mesenchymal cells. We are now also beginning to understand the mechanisms by which directed movement emerges. The mechanism of directed migration for the neural crest is supracellular, however, it remains an interesting and open question as to whether supraorganismal behaviour exists. Like in cells, the long-range direction can be imparted through communication between individual animals within a group, and leader and follower individuals can emerge [112]. In some animal groups, individuals do not take on specific roles of leaders and followers; instead, directed movement is imparted by each individual knowing how much local food there is [112]. Unlike in cells, animals do not have a physical structure that coordinates their directed movement; instead, group awareness and behaviour can emerge when organisms move more quickly in unfavourable environments; this modulation of speed as a function of local conditions allows the group to detect gradients even when any particular individual cannot [112]. These individual-to-individual behaviour differences result in fluid-like movement similar to that of supracells, which suggests some collective animal migration may be better understood at the level of the group. And in the case of animals, there is also growing evidence that migratory animals use social cues and that collective factors can shape movement [112].

The cellular mechanisms by which alignment arises are also unclear. Although CIL can contribute to cell alignment, whether this is the predominant, or only, mechanism by which alignment occurs is not known. Given alignment only constitutes a portion of the CIL response, it is likely that other methods of cell–cell communication contribute to aligned motion.

## Data accessibility

This article has no additional data.
